# Focused Ultrasound Enhances Targeted Gene Delivery of Intravenous AAV to the Brain

**DOI:** 10.1002/alz70859_103550

**Published:** 2025-12-25

**Authors:** Malik White, Rikke Han Kofoed, Chinaza Lilian Dibia, Nathalie Vacaresse, Luis Fernando Rubio‐Atonal, Brandy Laurette, Sheng‐Kai Wu, Sofia Samuelsson, Marie‐Eve Paquet, Kullervo Hynynen, Isabelle Aubert

**Affiliations:** ^1^ University of Toronto, Toronto, ON Canada; ^2^ Sunnybrook Research Institute, Toronto, ON Canada; ^3^ Center for Experimental Neuroscience (CENSE), Department of Neurosurgery, Aarhus University Hospital, Aarhus N, Denmark, Aarhus N, Aarhus Denmark; ^4^ Aarhus University, Aarhus, Jutland Denmark; ^5^ Aarhus Univresity Hospital, Aahrus, Jutland Denmark; ^6^ Sunnybrook Research Institute, toronto, ON Canada; ^7^ Faculty of Medicine ‐ University Laval, Quebec City, QC Canada; ^8^ Cervo Brain Research Institute, Quebec City, QC Canada; ^9^ Physical Sciences, Sunnybrook Research Institute, Toronto, ON Canada

## Abstract

**Background:**

Recombinant adeno‐associated viruses (AAVs) hold promise for brain disorders but often require intracranial injection, limiting therapeutic reach and being invasive. Intravenous (i.v.) AAV delivery is restricted by the blood‐brain barrier (BBB), reducing efficiency. Recent AAV variants can cross the BBB, enabling widespread brain transduction. MRI‐guided focused ultrasound (FUS) transiently modulates BBB permeability, enhancing gene delivery in small or large brain regions targeted.

**Hypothesis:**

We hypothesize that combining IV BBB‐penetrating AAVs with FUS will optimize gene delivery, enabling both widespread and region‐specific transduction for neurodegenerative diseases.

**Methods:**

Three‐month‐old C57BL/6 mice received i.v. AAV‐PHP.V1.CAG.TdTomato (V1) and AAV9.CAG.EYFP, followed by FUS targeting various brain regions. Immunohistochemistry and confocal microscopy were done 3 weeks post‐delivery.

**Results:**

Our findings demonstrate that FUS enhances targeted gene delivery for AAV9 and V1 while maintaining global expression in the brain for V1, offering a non‐invasive strategy for precise (AAV9) or widespread and locally enriched (V1) gene therapy.

**Conclusion:**

This approach could improve treatments for neurodegenerative disorders such as Alzheimer’s and Parkinson’s disease.

